# Biochemical, Biological, and Clinical Properties of γ-Oryzanol

**DOI:** 10.3390/antiox14091099

**Published:** 2025-09-09

**Authors:** Helena Juricic, Massimiliano Cuccioloni, Laura Bonfili, Mauro Angeletti, Daniela Uberti, Anna Maria Eleuteri, Giulia Abate, Valentina Cecarini

**Affiliations:** 1 School of Biosciences and Veterinary Medicine, University of Camerino, 62032 Camerino, Italy; helena.juricic@unicam.it (H.J.); massimiliano.cuccioloni@unicam.it (M.C.); laura.bonfili@unicam.it (L.B.); mauro.angeletti@unicam.it (M.A.); annamaria.eleuteri@unicam.it (A.M.E.); 2 Division of Pharmacology—Department of Molecular and Traslational Medicine, University of Brescia, 25123 Brescia, Italy; daniela.uberti@unibs.it

**Keywords:** γ-Oryzanol, rice bran, antioxidant, extraction techniques, bioavailability enhancement, biological activity

## Abstract

γ-Oryzanol is a complex mixture of ferulic acid esters of phytosterols and triterpene alcohols predominantly found in rice bran. It exhibits a wide range of biological activities, including antioxidant, anti-inflammatory, and lipid-lowering effects, as well as the ability to modulate cellular metabolic pathways in both in vitro and in vivo models. The composition and concentration of γ-oryzanol vary significantly among rice varieties and are influenced by genetic, environmental, and technological factors. Advances in extraction methods, including traditional solvent extraction and innovative approaches such as supercritical fluid extraction, have improved yield and purity, supporting its use in functional foods, nutraceuticals, and cosmetics. Current research in the biological, biomedical, and cosmetic fields is actively investigating γ-oryzanol’s mechanisms of action in metabolic regulation and inflammation, as well as developing advanced formulation strategies to enhance its antioxidant, skin-protective, and functional properties. These efforts aim to optimize its delivery and efficacy by addressing challenges related to poor water solubility and bioavailability, thereby expanding its role as a multifunctional bioactive compound. This review provides a comprehensive overview on γ-oryzanol, focusing on its extraction techniques, chemical characterization, and biological/pharmacological activities. Additionally, clinical trials investigating its efficacy and safety have been thoroughly dissected, offering valuable insights into its therapeutic potential in human populations.

## 1. Introduction

**Figure 1:**
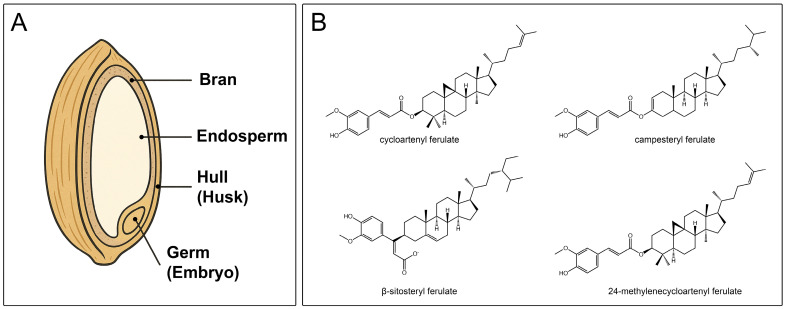
Schematic representation of rice grain structure (**A**), and chemical structure of the main γ-oryzanol components present in rice bran (**B**).

γ-Oryzanol is a natural bioactive compound that has received a lot of attention due to its biological properties and broad range of uses in food, cosmetics and pharmaceutical industries. It is present in wheat bran and some fruits and vegetables, with rice bran being the richest source (Figure 1, Panel A) [1]. Rice bran is the outer brown layer of the rice grain, and it consists of pericarp, aleurone, seed coat and germ. During the rice milling process, it is removed, leaving the polished white rice as the final product. Rice bran, as a nutrient-rich byproduct of rice production, consists of 50% carbohydrates, mostly starch, 20% fat, 15% protein, 15% fiber, and it further contains numerous bioactive components, including tocopherols, tocotrienols and oryzanol [2]. The biosynthesis of γ-oryzanol in rice bran occurs rapidly in the early phase of grain development which spans from 10 to 20 days after flowering, followed by slower increase until it reaches the peak levels at maturity (around 30 days after flowering) [3]. It was first isolated by Kaneko and Tsuchiya in 1954 from rice bran’s unsaponifiable fraction, receiving its name after the source from which it was obtained, *Oryza sativa* (rice) [4]. Previously thought to be a single compound, γ-oryzanol is now known to be a mixture of esterified sterols, ferulic acid and triterpene alcohols.

Physicochemically, γ-oryzanol is a white to off-white, odorless, and tasteless crystalline powder that is virtually insoluble in water but with a slight solubility in alkalinized methanol and ethanol. This behaviour is due to the weakly amphiphilic nature of γ-oryzanol, which features a phenolic hydroxyl group that acts as a weak acid, along with a long hydrophobic tail. In an alkaline environment, γ-oryzanol converts into γ-oryzanol salt, able to dissolve in methanol/ethanol [5]. Furthermore, γ-oryzanol is highly soluble in dimethyl sulfoxide (DMSO) because DMSO, acting as a polar aprotic solvent, interacts with polar esters and hydroxyl groups in γ-oryzanol via hydrogen bonding with its sulfoxide group. Moreover, the hydrophobic tail of γ-oryzanol is compatible with the two methyl groups of dimethyl sulfoxide, due to both favourable hydrogen bonding and van der Waals interactions.

Despite extensive evidence from in vitro and in vivo studies supporting its antioxidant and anti-inflammatory activities, its potential to lower plasma triglycerides and cholesterol levels and its ability to induce transcriptional factors [6,7,8], its broader application is hampered by challenges related to extraction and delivery. The extraction of γ-oryzanol is a crucial step for its utilization across various industrial sectors. Different strategies to improve the yield and purity during extraction have been developed including extraction with different solvents, liquid–liquid phase extraction, solid phase extraction, supercritical fluid extraction, microwave-assisted (MAE) and enzymatic–assisted extraction (EAE). Another major challenge with γ-oryzanol is its poor solubility in aqueous environments. For this reason, to perform its beneficial functions, it needs to be delivered in a form that is readily accessible to the organism. Therefore, studies that explore different innovative ways to increase its bioavailability and delivery success have been carried out further promoting the potential of this compound.

This review provides a comprehensive updated analysis on γ-oryzanol, including the methods of extraction, its biological and (potential) pharmacological functions, as well as the development of innovative delivery strategies.

## 2. Chemical Characterization and Composition of *γ*-Oryzanol

γ-Oryzanol was initially isolated in the 1950s, and shortly thereafter, it was determined to be a mixture of different components. Its main components are esters of trans-ferulic acids and plant sterols, which include more than 23 different steryl ferulates with cycloartenyl ferulate, 24-methylenecycloartanyl ferulate, campesteryl ferulates, and β-sitosteryl ferulates being mainly represented in most cultivars [9]. 24-methylenecycloartanyl ferulate and cycloartenyl ferulate are categorized as triterpene alcohol type, while β-sitosteryl ferulate and campesteryl ferulate are categorized as phytosterol type.

The work by Xu and Godber published in 1999 defined the chemical profile of γ-oryzanol [10], reporting the presence of major constituents such as cycloartenyl ferulate, 24-methylenecycloartanyl ferulate, and campesteryl ferulate. This study became then a cornerstone for subsequent research on γ-oryzanol and established a basis for its analysis in rice bran oil (Figure 1, Panel B).

In 2009, Khuwijitjaru et al. shifted the focus to the stability of γ-oryzanol. Their work on the degradation kinetics in rice bran oil provided critical insights into how this compound responds to environmental factors such as heat and oxidation. They identified key components, including cycloartenyl ferulate and campesteryl ferulate, and analysed their behaviour under various conditions [11]. This study greatly enhanced the understanding of γ-oryzanol’s stability and its potential for degradation.

The biological relevance of γ-oryzanol was further explored in 2012 by Saenjum, who highlighted the potent antioxidant properties of its major components. By identifying cycloartenyl ferulate, 24-methylenecycloartanyl ferulate, and campesteryl ferulate, the study highlighted the health-promoting effects of these bioactive compounds and their contribution to the biological significance of γ-oryzanol [12].

In 2018, Kour et al. developed an online coupled LC—GC method specifically for γ-oryzanol analysis in rice lipids [13]. This technique enabled the identification of prominent components, including cycloartenyl ferulate, 24-methylenecycloartanyl ferulate, campesteryl ferulate, and β-sitosteryl ferulate. Their work represents a critical reference for the detailed characterization of γ-oryzanol, further confirming the potential of LC-GC in lipid research. Sulaiman et al. in 2021 provided a comprehensive overview on γ-oryzanol, characterizing it as a mixture of phytosteryl ferulates [14]. Their study delved into its composition and biological activities, further reinforcing the compound’s multifunctional nature and its wide-ranging applications. This work not only expanded the understanding of γ-oryzanol’s structure but also emphasized its importance in various biological and industrial contexts. Most recently, in 2024, Vardhani et al. conducted a study on ethanolic extracts of Indonesian rice bran, offering new insights into γ-oryzanol’s structure and composition [15]. This research highlighted the bioactive potential of γ-oryzanol as a blend of ferulates and underscored the significance of regional rice varieties in contributing to its characterization and application. These findings are particularly relevant for evaluating the geographical diversity of γ-oryzanol sources.

γ-Oryzanol is primarily concentrated in the bran layer of rice grains and its total amount as well as ratio of its components varies depending on the origin, genotype and environmental factors. In a comparative study, Huang and Ng found that γ-oryzanol was significantly more concentrated in the bran layer compared to the endosperm, emphasizing the importance of the bran in determining the overall nutritional profile of rice [16]. A study by Minatel et al. indicated that coloured rice varieties, including black and red rice, tend to have higher γ-oryzanol content compared to white rice [17]. Another detailed study analysed γ-oryzanol content in several rice varieties, mostly long grain types like Basmati, and found significant variations. In details, it was shown that Basmati varieties had higher γ-oryzanol content (up to 330.3 mg/kg in Basmati Pak) compared to other cultivars, with total γ-oryzanol ranging from about 246.7 to 330.3 mg/kg of brown rice seed [18]. This suggests that the type of rice—round or long grain—can influence the levels of this beneficial compound. In addition to that, the extraction methods used to obtain rice bran oil can also affect the γ-oryzanol content. Sawadikiat & Hongsprabhas noted that different extraction techniques yield different amounts of γ-oryzanol, with some methods preserving more of this compound than others [19]. They demonstrated that crude rice bran oil contains approximately 1362–1376 mg/100 g of phytosterols and 1599–1666 mg/100 g of γ-oryzanol whereas physical refining process significantly reduces the content of these bioactives, with refined rice bran oil containing only about 820–895 mg phytosterols and 933–960 mg γ-oryzanol per 100 g [19].

Research on different pigmented rice also highlighted the difference in γ-oryzanol composition among rice varieties. Tsuzuki et al. have compared γ-oryzanol profiles of black-purple, red, brown and green varieties of rice distributed in Japan. Black-purple rice showed significantly higher content of γ-oryzanol as well as partially specific composition of steryl ferulates compared to the red, brown and green varieties [20]. The analysis of red, black, brown and white rice from the Camargue region of France revealed the different γ-oryzanol contents as 79, 63, 12 and 8.2 mg/g, respectively. Four major components were also detected, 24-methylenecycloartanyl ferulate (27–38%), cycloartenyl ferulate (23–41%), campesteryl ferulate (19–31%) and β-sitosteryl ferulate (7–20%) [21]. Similarly, 16 rice varieties from Korea, among which 5 pigmented, were reported to contain 19 different γ-oryzanol components which were identified as stigmasterol, campesterol and sitosterol and common triterpenoids, with γ-oryzanol levels ranging from 26.7 to 61.6 mg/100 g of rice [22].

Globally, while γ-oryzanol is a valuable component found predominantly in rice bran, its concentration can vary widely between different rice varieties, including round and long grain types. Factors such as genetic variability, environmental conditions, and extraction methods play crucial roles in determining the levels of γ-oryzanol in rice products and a deep understanding of their influence can help to maximize the health benefits associated with γ-oryzanol-rich rice bran products.

## 3. Extraction Techniques

The extraction of γ-oryzanol from rice bran has attracted considerable interest due to its several health benefits, and in this context a number of extraction methods have been developed and optimized to isolate this bioactive compound, each with distinct advantages and challenges. Table 1 summarizes the methods currently used for γ-oryzanol extraction. Previously described extraction methods embrace direct solvent extraction, liquid–liquid phase extraction, solid phase extraction, supercritical fluid extraction, microwave-assisted (MAE) and enzymatic–assisted extraction. Solvent extraction remains the most widely employed technique due to its simplicity and efficiency. Traditional solvents such as hexane, ethanol, and acetone are commonly used for γ-oryzanol extraction [23]. Chen et al. proposed a rapid and convenient equilibrium extraction method using methanol as a solvent coupled with RP-HPLC [24,25]. Although solvent extraction is efficient in terms of yield, the use of large quantities of organic solvents raises environmental concerns, as well as the need for solvent recovery and disposal [26]. To address these concerns, supercritical fluid extraction (SFE) has emerged as a promising alternative. This technique usually utilizes carbon dioxide (CO_2_) as a solvent due to its low cost, non-combustibility, and its safety in both foods and environment [27]. SFE has demonstrated the ability to extract γ-oryzanol with high yields and minimal residual solvent contamination. Recent findings demonstrated that the yield of oryzanol by SFE is higher than that obtained by solvent extraction conducted with different combinations of solvents and processing conditions. For example, Imsanguan et al. performed and compared different extraction methods for γ-oryzanol from rice bran including SC-CO_2_ and solvent extraction with hexane and ethanol. They indicated that SC-CO_2_ gave the best results in terms of extraction (48 MPa, 65 °C) with a yield of 11.4 mg/kg (dry basis) [28]. Xu et al. compared SC-CO_2_ and solvent extraction methods obtaining that the yield of γ-oryzanol (5.39 mg/g of rice bran) in SFE (temperature 50 °C, pressure of 68.901 kPa (680 atm), and time of 25 min) was approximately four times higher than the highest yield of solvent extraction (1.68 mg/g of rice bran) that was performed with a solvent mixture with 50% hexane and 50% isopropanol (*v*/*v*) (temperature 60 °C, 45–60 min) [23]. However, SFE still finds limited application mainly because of the high associated costs and operational complexity considering that it requires precise control of pressure and temperature [29].

To further enhance extraction efficiency and reduce solvent usage, microwave-assisted extraction (MAE) has also been explored. This technique uses microwave radiation to heat the solvent, facilitating better penetration of the solvent into the rice bran matrix and accelerating the extraction process. Kumar et al. obtained high concentration of γ-oryzanol upon methanolic extracts of microwave treatment compared to ultrasonication and to conventional extraction method. In details, they indicated that a combination of 80% methanol concentration and 55 min digestion time of microwave treatment yielded the best extraction for γ-oryzanol (105 ppm) [30].

Enzyme-assisted extraction (EAE) and aqueous enzymatic extraction (AEE) are widely used sustainable and eco-friendly processes. Specific enzymes are used to break down cell walls in rice bran, thereby facilitating the release of oil into the aqueous system while minimizing the need for harsh solvents and high temperatures. This method has been shown to produce high yields while also being more selective in terms of the bioactive compounds extracted. A combination of cellulase and alcalase (1:1, *w*/*w*) was found to be the best strategy for the extraction of rice germ oil, with a good γ-oryzanol content, allowing the destruction of the structural integrity of the rice germ cell wall [31].

Loypimai et al. introduced an innovative green extraction method using ultrasound techniques with soybean oil. This process improves the functionality of edible vegetable oils and produces vegetable oils containing high bioactive substances without the necessity of separating environmentally hazardous organic solvents [32]. Ultrasound assisted extraction combined with D-limonene as solvent was optimal for extracting γ-oryzanol from rice berry bran [33].

Despite the advances in extraction technologies, challenges still exist in optimizing the extraction conditions to balance high yield, minimal environmental impact, and cost-efficiency. Factors such as solvent choice, extraction temperature, pressure, and time must be carefully adjusted to ensure the maximum recovery of γ-oryzanol, that is extremely variable among different sources, while maintaining the stability of the compound. Furthermore, the scalability of these extraction methods from the laboratory to industrial levels remains a key consideration. In this perspective, while traditional solvent extraction methods remain widely used, novel, cost-effective and sustainable extraction techniques such as supercritical fluid extraction, microwave-assisted extraction, and enzymatic and green methods are gaining attention.

**Table 1:** Summary of extraction methods for γ-oryzanol.

Technique	Description	Advantages	Limitations/Challenges
Solvent Extraction [23,24,25,26]	Use of solvents such as hexane, ethanol, acetone, conventional and widely used method	Simple, efficient, high yield	Large solvent volumes, environmental concerns, solvent disposal
Supercritical Fluid Extraction (SFE) [27,28,29]	Use of supercritical CO_2_ under controlled temperature and pressure for extraction	High purity, high yield, minimal solvent residue	High operational cost, complexity of pressure and temperature control
Microwave-Assisted Extraction (MAE) [30]	Microwave radiation heats solvent to enhance extraction efficiency	Increased extraction yield, reduced extraction time	Optimization of microwave parameters
Enzyme-Assisted Extraction (EAE) and Aqueous Enzymatic Extraction (AEE) [31]	Use of enzymes (e.g., cellulase, alcalase) to degrade cell walls and release oil	Eco-friendly, selective extraction, high yield	Enzyme cost, optimization of enzymatic conditions
Ultrasound-Assisted Extraction (UAE) [33]	Use of ultrasound waves with solvents or oils (e.g., soybean oil, D-limonene) to improve extraction	Green method, enhancement of bioactive compound yield	Equipment cost, scale-up challenges

## 4. Strategies for γ-Oryzanol Delivery

The poor water solubility of γ-oryzanol presents significant challenges for its absorption and bioavailability, limiting its application in food, pharmaceutical, and medical systems. Due to its hydrophobic nature means γ-oryzanol is not readily absorbed in the intestine, and conventional formulations often fail to achieve effective solubilization and therapeutic concentrations. To address this challenge, the use of surfactants, cosolvents, or advanced delivery technologies is necessary. Co-solvent strategies and surfactants for γ-oryzanol delivery enhance the solubility, stability, and bioavailability of γ-oryzanol by facilitating its incorporation into emulsions, self-emulsifying drug delivery systems, and nanocarriers.

Kozuka C. et al. encapsulated γ-oryzanol in polymer poly (DL-lactide-co-glycolide) (PLGA) nanoparticles and evaluated its impact on obese-diabetic *ob/ob* mice observing amelioration of both glucose and lipid metabolism [34]. Jasim A. et al. developed and characterized liposome nanocarriers incorporating γ-oryzanol with interesting antioxidant and hepatoprotective effects [35]. These new liposome nanocarriers containing γ-oryzanol offer a promising alternative for the advancement of drug delivery systems and therapeutic treatments. γ-Oryzanol-enriched nanoemulsions using fish oil and medium-chain triglyceride as carrier oils were produced by a low-energy emulsification method showing good stability in different physical conditions. These nanoemulsions could be interesting preparations for food systems, personal care and pharmaceutical products [36]. Rodsuwan et al. encapsulated γ-oryzanol into zein (the prolamine protein from corn endosperm) nanoparticles obtaining good stability with the zein coating providing the control release of γ-oryzanol in a simulated gastrointestinal fluid [37].

Increasing interest has been focused on the development of self-emulsifying drug delivery systems (SEDDS formulations). Solid self-nanoemulsifying drug delivery systems (S-SNEDDS) were shown to successfully improve oral delivery of γ-oryzanol with a 96% drug loading efficiency [38]. Self-emulsified alginate beads (SEABs) loaded with γ-oryzanol and algae oil were developed and shown to be an effective controlled delivery system. In an in vitro release study, these beads successfully passed through the simulated stomach fluid without releasing their contents, then were dissolved in the intestinal fluid, where they effectively released oryzanol [39].

Briefly, various delivery strategies for γ-oryzanol have been developed to overcome problems such as low water solubility, poor bioavailability, and susceptibility to degradation (Table 2). These advanced delivery systems not only protect γ-oryzanol from degradation in the gastrointestinal tract but also enable targeted and sustained release, thereby maximizing its therapeutic potential. Such innovations hold promise for the effective application of γ-oryzanol in functional foods and pharmaceutical formulations. Moreover, exploring the mechanisms of controlled release, biodistribution, long-term safety, and therapeutic outcomes will be critical to improve these nanodelivery systems from experimental stages to clinical use. Future research should integrate formulation optimization, toxicological profiling, and efficacy testing to fully realize the clinical benefits of γ-oryzanol delivery strategies.

## 5. Biological Functions of γ-Oryzanol

**Figure 2:**
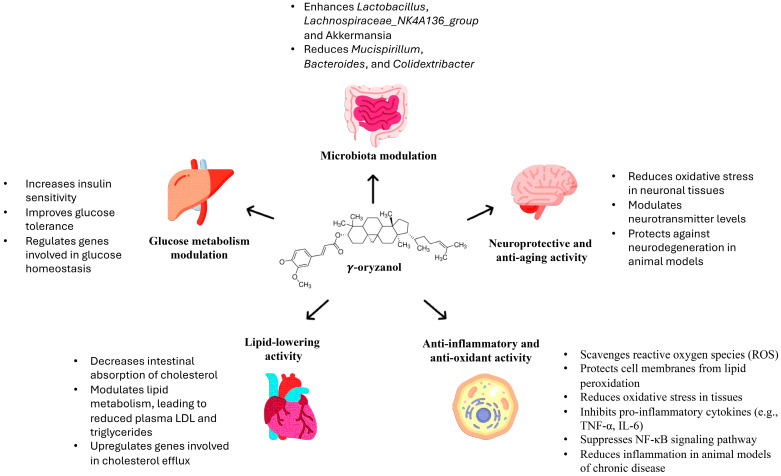
Main targets and biological effects exerted by γ-oryzanol.

γ-Oryzanol has been reported to possess numerous beneficial properties including antioxidant, anti-inflammatory, anti-aging, cholesterol-lowering, and neuroprotective effects (Figure 2). All these properties suggest γ-oryzanol as a valuable multitarget ingredient for pharmaceutical and cosmetic formulations and for foods.

Using HPLC-MS and HPLC-MS/MS techniques, Kobayashi E. et al. demonstrated that γ-oryzanol compounds were absorbed and remained in their intact form in plasma following administration of rice bran oil to mice. Consequently, the substantial presence of intact γ-oryzanol in vivo may account for its strong beneficial effects [40]. The same group analysed γ-oryzanol presence in different organs upon rice bran oil and rice bran oil containing a high concentration of γ-oryzanol long-term administration. γ-Oryzanol was detected in all the analysed organs (liver, brain, kidney, spleen, muscle, mesenteric fat, and perirenal fat), mainly in the liver, in both groups, suggesting that γ-oryzanol, once ingested, is widely distributed in the mice body [41]. Moreover, a lower plasma lipid concentration was detected in mice supplemented with rice bran oil containing a high concentration of γ-oryzanol. According to the authors, additional analyses of its metabolites will provide a deeper insight into its metabolism and biological effects. Nonetheless, these findings indicate that γ-oryzanol targets various organs in the body, leading to a range of biological activities.

Numerous data have elucidated both the in vitro and in vivo antioxidant properties of γ-oryzanol. These observations revealed that the antioxidant and anti-inflammatory activities are exerted via radical oxygen species (ROS) scavenging and inhibition of ROS production. It has been demonstrated that the activity of the major γ-oryzanol components was significantly higher than that of the vitamin E components (both extracted from rice bran) in inhibiting cholesterol oxidation [42]. Additional data showed that phytosteryl ferulates such as cycloartenyl ferulate, 24-methylenecycloartanyl ferulate, and β-sitosteryl ferulate and ferulic acid exerted strong free radical scavenging and antioxidation of lipid membrane, which were comparable to α-tocopherol [43]. Juliano et al. indicated its ability as a radical scavenger able to prevent lipoperoxidation processes thus having a potential application as a natural antioxidant for the stabilization of lipidic raw materials [44]. The antioxidant effects of γ-oryzanol were also evaluated in a model of Parkinson’s disease induced by the exposure of *D. melanogaster* to rotenone. γ-Oryzanol was able to reduce the deleterious effects of rotenone showing a neuroprotective action due to its antioxidant constituents such as ferulic acid [45]. γ-Oryzanol was also found to enhance skeletal muscle function and to improve oxidative capacity in aged mice by regulating PPARδ and ERRγ activity without muscle gain [46].

Dietary γ-oryzanol plays a significant role in the anti-inflammatory activity of rice bran oil by decreasing pro-inflammatory mediators secreted by rat peritoneal macrophages [47]. γ-Oryzanol administration in a mouse model of colitis induced by dextran sulphate sodium markedly inhibited inflammatory reactions including myeloperoxidase activity, release of pro-inflammatory cytokines and COX-2 levels possibly via the inhibition of NF-κB activity through scavenging reactions of free radicals [48]. Mattei L. et al. reported that the treatment with γ-oryzanol improved insulin resistance, reduced inflammation, increased antioxidant response and glucose transporter-4 (GLUT-4) expression in skeletal muscles of obese animals [49]. Both the antioxidant and anti-inflammatory activity of γ-oryzanol contributed to attenuate insulin resistance by increasing GLUT-4 expression in the skeletal muscle of obese animals [49]. By reducing inflammation and oxidative stress, γ-oryzanol also contributes to skin protection, tissue regeneration, and barrier maintenance. For example, advanced formulations made of γ-oryzanol-loaded nanoethosomes (GO-NEs) prevent UVB-induced skin cancer by enhancing antioxidant activity [50]. Studies have demonstrated the possibility to incorporate γ-oryzanol into advanced cosmetic formulations such as nanogels and nanoethosomes, showing skin protection and compatibility [51]. These multifunctional effects thus support its use in a variety of cosmetic products aimed at favouring skin protection and health.

γ-Oryzanol exhibits also a strong capacity to modulate lipid metabolism and reduce cholesterol levels through several mechanisms including the inhibition of gastrointestinal cholesterol absorption, the increase in fecal excretion of bile acids and the inhibition of 3-hydroxy-3-methylglutaryl coenzyme A (HMG-CoA) reductase, an enzyme involved in cholesterol synthesis [52]. Animal studies have shown that dietary supplementation with γ-oryzanol leads to significant reductions in plasma lipid levels [31,41,53,54]. Interestingly, Yan S. et al. demonstrated that γ-oryzanol may affect serum lipid levels by regulating amino acid metabolism through gut microbiota modulation [55]. γ-Oryzanol and ferulic acid suppressed high fat-induced hyperlipidemia in mice increasing faecal excretion of cholesterol and triglyceride and inhibiting fatty acid biosynthesis. In addition, they mitigated oxidative stress induced by the high fat diet by enhancing the activity of antioxidant enzymes in the liver [56]. A recent interesting study demonstrates that γ-oryzanol alleviates metabolic dysfunction-associated steatohepatitis (MASH) in mice by reducing lipid accumulation and inflammation through different targets. They observed the modulation of gut microbiota, with increased *Lactobacillus*, *Lachnospiraceae_NK4A136_group,* and Akkermansia, and decreased harmful bacteria such as *Mucispirillum*, *Bacteroides*, and *Colidextribacter*, the enhancement of the gut barrier integrity, and a reduced endotoxemia and TLR4/NF-κB signalling pathway [57]. Additionally, we have obtained preliminary data indicating that γ-oryzanol can protect the intestinal epithelium from cytokine-induced damage using Caco-2 cell monolayers as an in vitro model (unpublished data). Together these data indicate that γ-oryzanol has a robust ability to modulate lipid metabolism and protect from abnormal gut permeability through diverse and complementary mechanisms, supporting its potential as a natural therapeutic agent for managing hyperlipidemia and metabolic syndrome.

γ-Oryzanol also exhibits significant neuroprotective effects through multiple mechanisms. Studies confirm that γ-oryzanol crosses the blood–brain barrier without structural modification, exhibiting high distribution levels within the brain [58]. It has been shown that it antagonizes glutamate-induced excitotoxicity in an in vitro model of differentiated HT-22 neuronal cells reducing oxidative stress, preventing the loss of mitochondrial membrane potential, and lowering calcium overload [59]. Additionally, it prevents glutamate-mediated apoptosis decreasing calcium/calmodulin-dependent protein kinase II activation to block the ASK-1/c-Jun/AP-1 cascade [59]. Recently, we demonstrated that γ-oryzanol can modulate neurite outgrowth and exert a neurotrophic effect, potentially through the induction of the Nrf2 nuclear translocation and activation of Nrf2-ARE phase II enzymes [60]. Additionally, quantitative changes in proteins involved in synaptic plasticity and neuronal trafficking, neuroprotection and antioxidant activity, mitochondria and energy metabolism were observed suggesting that it can help preserving brain function [61]. The promising neuroprotective action of γ-oryzanol was also indicated by another work showing its anxiolytic, neurogenic, and anti-neuroinflammatory properties in mice under chronic consumption of 10% ethanol [62]. These findings suggest that γ-oryzanol could be a promising candidate for the development of therapeutic strategies to counteract aging and neurodegenerative diseases.

γ-Oryzanol can also modulate endocrine function influencing hormone levels, modulating sex hormones and neurotransmitters, thereby supporting balanced endocrine and neurological activity. It counteracts perimenopausal symptoms through different mechanisms, by enhancing neurotransmitter release, promoting osteoblast proliferation via upregulation of bone formation genes to increase bone mineral density and prevent osteoporosis, and improving sleep quality by inhibiting the histamine H1 receptor, which shortens sleep latency and encourages non-rapid eye movement sleep [63,64,65,66]. This multifaceted action thus supports endocrine balance, bone health, and better sleep during perimenopause. It is also indicated for attenuating menopausal depression since it reduces depressive behaviour in ovariectomized mice regulating hippocampal nitric oxide synthase [67]. Co-administration of γ-aminobutyric acid and γ-oryzanol recovered the adiponectin levels in stress-induced hypoadiponectinemia mice into normal levels suggesting a role in metabolic syndromes improvement [68]. Kozuka et al. demonstrated that γ-oryzanol alleviates endoplasmic reticulum (ER) stress-induced β-cell dysfunction and prevents subsequent apoptosis, underscoring the potential of γ-oryzanol as a therapeutic agent for diabetes mellitus [69]. γ-Oryzanol from brown rice also improved glucose dysmetabolism and attenuated the preference for dietary fat in mice fed an high fat diet by decreasing hypothalamic ER stress and through the epigenetic modulation of dopamine D2 receptors in brain striatum [70,71]. These findings suggest that brown rice and its extracts may be useful tools for ameliorating obesity and metabolic syndrome.

## 6. Effect on Redox Enzymes

γ-Oryzanol has been shown to exert a context-sensitive modulation of cellular antioxidant defences, functioning as either a suppressor or enhancer depending on the physiological or pathological status of the tissue.

In pathological contexts such as cancer or persistent oxidative stress, γ-oryzanol downregulates redox enzymes, most notably catalase (CAT), glutathione peroxidase (GPX), and superoxide dismutase (SOD) [72]. This downregulation weakens the cell’s natural defence mechanisms against reactive oxygen species (ROS), leading to increased oxidation within the cell. Mechanistically, when γ-oryzanol inhibits catalase, intracellular hydrogen peroxide accumulates, while reduction in GPX activity impairs the conversion of lipid hydroperoxides and H_2_O_2_ into less reactive species, collectively amplifying both oxidative stress and the cell’s susceptibility to programmed cell death or cytotoxic therapies. These alterations are often coupled with a reduction of cellular glutathione (GSH), further depriving the cell of another major intracellular antioxidant line of defence and shifting the redox balance toward conditions favourable for ROS-mediated tumour cell death. Evidence from mammalian models confirms that this concerted enzyme downregulation and GSH depletion can sensitize cancerous cells to ROS-dependent apoptosis, enhancing the efficacy of chemotherapeutic interventions that rely on redox mechanisms.

In contrast, in non-pathological (healthy) tissues or models of organ injury, neurodegeneration, and systemic inflammation, γ-oryzanol strongly enhances the activities of endogenous antioxidant enzymes (CAT, GPX, SOD) and increases GSH levels [73]. In healthy tissues, this supports overall cellular redox stability and homeostasis, mitigating the potential damage from basal or stress-induced ROS generation. Specifically, in rodent models subjected to ischemia–reperfusion or sepsis, γ-oryzanol administration significantly increased hepatic antioxidant enzyme activities and tissue GSH, concurrently decreasing both lipid peroxidation markers such as malondialdehyde (MDA) and protein oxidative damage, which are key indicators of oxidative injury. Similarly, data from Drosophila models of neurodegeneration show that γ-oryzanol supplementation counteracts pesticide-induced ROS accumulation and oxidative injury, mainly through upregulated CAT, SOD, and glutathione S-transferase (GST) activities, resulting in lower ROS burden and reduced lipid peroxidation [74].

At the molecular level, γ-oryzanol’s dual redox regulation is shaped by its capacity to interact directly with radical species and to regulate gene expression pathways associated with cellular antioxidant response. In vitro, γ-oryzanol acts as a potent organic radical scavenger, directly neutralizing free radicals such as those generated by the thermal decomposition of AMVN (a lipid-soluble initiator of lipid peroxidation), and preventing the chain propagation of lipid peroxidation [44]. This effect demonstrates dose-dependency and is especially notable for oils and lipid-rich systems susceptible to oxidative deterioration, with γ-oryzanol stabilizing such substrates comparably to synthetic antioxidants like butylated hydroxytoluene (BHT) at higher concentrations. Mechanistically, in addition to its direct free radical scavenging action, γ-oryzanol modulates several signalling pathways including Nrf2/ARE activation (which promotes broad antioxidant gene expression), NF-κB suppression (inhibiting pro-inflammatory and pro-oxidant gene networks) [75], attenuation of the AGEs/RAGE axis [76], and induction of phase II antioxidant genes including NAD(P)H:quinone oxidoreductase 1 (NQO1) and heme oxygenase-1 (HO-1) contributing to both cytoprotective and anti-inflammatory effects [77].

**Figure 3:**
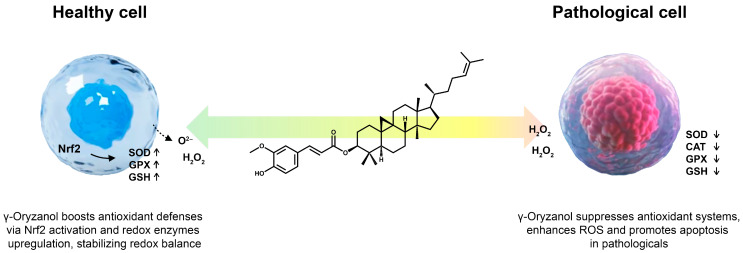
Environment-dependent redox properties of γ-oryzanol.

Due to these environment-dependent, bidirectional roles, γ-oryzanol can act as a disease-modifying agent, promoting cell survival and redox stability in healthy or inflamed tissues but fostering ROS accumulation and enhancing cell death in pathological environments such as cancer or chronic oxidative injury (Figure 3). This duality highlights γ-oryzanol’s potential for targeted therapeutic strategies, either to sensitize malignant cells to oxidative damage or to protect vulnerable tissues from redox imbalance and inflammation.

## 7. Clinical Applications and Therapeutic Evidence of γ-Oryzanol

γ-Oryzanol has a long clinical history, originating in Japan during the mid-1950s when it was first isolated from rice bran. The compound obtained pharmaceutical approval in Japan by 1962, initially for anxiety management and subsequently expanded to include menopausal symptoms, elevated cholesterol levels, and gastric disorders [78]. This early regulatory acceptance established γ-oryzanol as a legitimate therapeutic agent well before extensive contemporary clinical validation frameworks were established. Since then, the therapeutic acceptance of γ-oryzanol has since extended globally, with the compound achieving U.S. FDA GRAS (Generally Recognized as Safe) certification in 2019, facilitating its incorporation into food products and dietary supplements. Likewise, European regulatory authorities have approved γ-oryzanol use in functional foods and supplements, confirming its safety profile and therapeutic potential.

γ-Oryzanol is widely recognized for cardiovascular benefits, particularly in managing lipid abnormalities. Clinical studies reveal modest yet significant cholesterol-lowering effects, with evidence showing that supplementation can reduce total cholesterol, LDL cholesterol, and triglyceride levels in hypercholesterolemic patients [6]. A randomized, double-blind, placebo-controlled study demonstrated remarkable results with combined supplementation, achieving a 19.3% reduction in LDL-C and a substantial 29.3% increase in HDL-C over three months in adults with mild dyslipidemia [79]. These lipid modulations align with the mechanistic actions of γ-oryzanol, which include inhibition of cholesterol absorption and HMGCR activity, alongside regulation of lipid metabolism pathways [52,53,54,55].

Beyond lipid lowering, γ-oryzanol exerts pleiotropic effects on arterial health by influencing inflammatory and oxidative pathways involved in atherosclerosis progression, highlighting its multi-target therapeutic potential in cardiovascular medicine. Clinical evidence supports γ-oryzanol’s utility in metabolic syndrome management, where it improves insulin sensitivity, enhances glucose metabolism, and modulates adipokine profiles [80,81]. Studies involving patients with type 2 diabetes have shown that daily γ-oryzanol intake reduces systemic inflammatory markers such as C-reactive protein (CRP), interleukin-6 (IL-6), and interferon-gamma (IFN-γ), indicating its anti-inflammatory efficacy in metabolic contexts [82]. These clinical findings complement the molecular anti-inflammatory and antioxidant mechanisms, behind improved metabolic homeostasis.

γ-Oryzanol has demonstrated clinical benefits in alleviating menopausal symptoms. Historical data from Japanese studies found that daily administration of 300 mg improved symptoms in over two-thirds of menopausal women within 38 days, with later studies reporting efficacy rates up to 85% [83,84]. The compound’s endocrine-modulating effects, including neurotransmitter regulation and bone health support through osteoblast proliferation, provide a mechanistic foundation for these clinical observations [62,64,65,66].

The anti-inflammatory potential of γ-oryzanol has been corroborated by clinical interventions in chronic diseases. In a 12-week randomized controlled trial involving type 2 diabetes patients, consumption of γ-oryzanol-fortified canola oil resulted in significant reductions in key inflammatory biomarkers, reinforcing its systemic anti-inflammatory capacity [82]. These clinical outcomes mirror the documented in vitro and in vivo suppression of NF-κB signaling and cytokine inhibition described in earlier sections.

The applications of γ-oryzanol for athletic performance remain inconclusive. While a well-controlled study by Fry and colleagues found no significant effects on performance measures or hormone concentrations in moderately weight-trained males receiving 500 mg daily over 9 weeks [83], a subsequent investigation demonstrated that 600 mg daily during 9-week resistance training increased muscular strength in young healthy males, though without changes in anthropometric measurements [81,85]. These contrasting findings underscore the need for further rigorous randomized studies to clarify its role and potential applications in sports medicine.

Emerging neuroprotective preclinical research suggests γ-oryzanol’s potential in neurodegenerative diseases, including Alzheimer’s and Parkinson’s disease [60,86]. It is capable of crossing the blood–brain barrier in intact form and exerts antioxidant and anti-inflammatory effects within the central nervous system. Limited clinical data, including cognitive improvements observed in elderly Japanese cohorts following dietary interventions containing γ-oryzanol, support its capacity to preserve neuronal function [87]. These corroborate mechanistic insights of neuronal differentiation and mitochondrial protection as detailed in previous studies [59,60,61].

A significant advantage of γ-oryzanol is its exceptional safety profile. Decades of clinical use have not identified any serious adverse effects, even at daily dosages up to 800 mg [88]. This safety dosage makes γ-oryzanol particularly suitable for long-term use in sensitive populations, including the elderly and those with comorbid chronic illnesses, addressing a critical unmet need for mild yet effective multi-target agents.

Despite promising data, current clinical studies have limitations. Most studies involve relatively small sample sizes and short treatment durations, typically ranging from 4–12 weeks, limiting insights into long-term efficacy and safety. Additionally, heterogeneous formulations using rice bran oil complexes rather than standardized γ-oryzanol preparations create challenges in attributing specific effects to γ-oryzanol versus other bioactive rice bran components. Standardization of γ-oryzanol preparations and larger, extended clinical trials are imperative to confirm and extend current findings.

Globally, γ-oryzanol exhibits considerable therapeutic potential validated by clinical evidence across cardiovascular, metabolic, menopausal, neuroprotective, and anti-inflammatory domains, supported by a robust safety record (Table 3). While mechanistic studies have elucidated its molecular actions, ongoing and future well-designed clinical trials with standardized compounds are essential for firmly establishing its role in modern therapeutic regimens.

## 8. Conclusions and Future Prospectives

γ-Oryzanol is a multitarget bioactive compound predominantly obtained from rice bran, showing a complex composition and a broad spectrum of health-promoting properties, including antioxidant, anti-inflammatory, and lipid-lowering effects. Additionally, recent evidence suggests that γ-oryzanol may influence the composition and activity of the gut microbiota, highlighting a novel mechanism underlying its health benefits. Advances in extraction technologies—ranging from traditional solvent-based methods to innovative green and supercritical fluid extraction—have significantly improved both the yield and purity of γ-oryzanol, enabling its incorporation into functional foods, nutraceuticals, and cosmetic products. Despite these achievements, challenges such as poor aqueous solubility and limited bioavailability remain, driving ongoing research toward the finding of novel delivery systems and formulation strategies to enhance its therapeutic potential. Future studies should prioritize the development of sustainable and cost-effective extraction methods alongside large-scale clinical trials to robustly confirm the efficacy and safety of γ-oryzanol in human populations. Preliminary clinical evidence has demonstrated that γ-oryzanol supplementation may contribute to improvements in lipid profiles, reduce oxidative stress markers, and alleviate symptoms related to inflammation and metabolic disorders. However, further investigations are necessary to establish standardized dosing protocols, evaluate long-term safety, and determine the scope of benefits across diverse populations. This will help consolidate γ-oryzanol’s role as a valuable ingredient in promoting human health and wellness.

## Figures and Tables

**Table 2:** Major delivery systems for γ-oryzanol.

Delivery System	Description	Benefits	Limitations/Challenges
Polymer Nanoparticles (PLGA) [34]	Encapsulation of γ-oryzanol in biodegradable polymer nanoparticles for metabolic disease models	Improved glucose and lipid metabolism in obese-diabetic mice	Requires complex formulation and characterization
Liposome Nanocarriers [35]	Incorporation of γ-oryzanol into liposomes for enhanced antioxidant and hepatoprotective effects	Enhanced stability and bioavailability	Stability and scale-up challenges
Nanoemulsions [36]	γ-Oryzanol nanoemulsions using fish oil and medium-chain triglycerides as carrier oils	Good physical stability, suitable for food, personal care, pharmaceuticals	Formulation optimization needed
Protein Nanoparticles (Zein) [37]	Encapsulation in zein protein nanoparticles for controlled release in gastrointestinal fluids	Controlled release profile, improved stability	Protein source variability can affect properties
Self-Nanoemulsifying Drug Delivery Systems (S-SNEDDS) [38]	Solid formulations enhancing oral delivery efficiency and drug loading	High drug loading (~96%), improved oral bioavailability	Formulation complexity, excipient compatibility
Self-Emulsified Alginate Beads (SEABs) [39]	Alginate beads loaded with γ-oryzanol and algae oil for controlled intestinal release	Protection in stomach fluid, effective intestinal release	Manufacturing and reproducibility

**Table 3:** Clinical applications and therapeutic evidence of γ-oryzanol.

Therapeutic Area	Evidence & Effects	Clinical Outcomes	Limitations/Future Needs
Cardiovascular and Lipid Metabolism [6,52,53,54,55,79]	Modest cholesterol reduction, decrease in LDL-C, total cholesterol, triglycerides, increase in HDL-C	19.3% LDL-C reduction, 29.3% HDL-C increase in mild dyslipidemia	Larger trials, standardized γ-oryzanol preparations needed
Metabolic Syndrome and Diabetes [80,81,82]	Amelioration of insulin sensitivity and glucose metabolism, reduction of inflammatory markers (CRP, IL-6, IFN-γ)	Improved metabolic biomarkers in type 2 diabetes	Long-term studies, mechanism elucidation required
Menopausal Symptoms [83,84]	Alleviation of symptoms via endocrine modulation, improvement of bone density and sleep quality	Symptom improvement in > 65–85% of women in clinical studies	Larger RCTs for dosing and safety
Neuroprotection and Cognitive Health [60,86,87]	It crosses the blood–brain barrier, antioxidant, anti-inflammatory and neurogenic effects	Cognitive improvements noted in elderly cohorts	More clinical trials in neurodegenerative diseases
Anti-inflammatory Effects [82]	Suppression of the NF-κB pathway, reduction of pro-inflammatory cytokines and oxidative stress	Reduced inflammation in chronic disease clinical trials	Further mechanistic and long-term clinical validation
Safety Profile [88]	Excellent safety profile, no serious adverse events at doses up to 800 mg/day	Safe for long-term use, elderly, chronic conditions	Continue monitoring in diverse populations
